# Graph theoretical analysis and independent component analysis of diabetic optic neuropathy: A resting‐state functional magnetic resonance imaging study

**DOI:** 10.1111/cns.14579

**Published:** 2024-03-18

**Authors:** Qian Wei, Si‐Min Lin, San‐Hua Xu, Jie Zou, Jun Chen, Min Kang, Jin‐Yu Hu, Xu‐Lin Liao, Hong Wei, Qian Ling, Yi Shao, Yao Yu

**Affiliations:** ^1^ Department of Endocrine and Metabolic The First Affiliated Hospital, Jiangxi Medical College, Nanchang University, Jiangxi Clinical Research Center for Endocrine and Metabolic Disease, Jiangxi Branch of National Clinical Research Center for Metabolic Disease Nanchang Jiangxi China; ^2^ Queen Mary School The Nanchang University Nanchang Jiangxi China; ^3^ Department of Radiology Xiamen Cardiovascular Hospital of Xiamen University, School of Medicine, Xiamen University Xiamen Fujian China; ^4^ Department of Ophthalmology The First Affiliated Hospital, Jiangxi Medical College, Nanchang University Nanchang Jiangxi China; ^5^ Department of Ophthalmology and Visual Sciences The Chinese University of Hong Kong Hong Kong China; ^6^ Department of Ophthalmology Eye & ENT Hospital of Fudan University Shanghai China

**Keywords:** diabetic optic neuropathy, graph theoretical analysis, independent component analysis, rs‐fMRI

## Abstract

**Aims:**

This study aimed to investigate the resting‐state functional connectivity and topologic characteristics of brain networks in patients with diabetic optic neuropathy (DON).

**Methods:**

Resting‐state functional magnetic resonance imaging scans were performed on 23 patients and 41 healthy control (HC) subjects. We used independent component analysis and graph theoretical analysis to determine the topologic characteristics of the brain and as well as functional network connectivity (FNC) and topologic properties of brain networks.

**Results:**

Compared with HCs, patients with DON showed altered global characteristics. At the nodal level, the DON group had fewer nodal degrees in the thalamus and insula, and a greater number in the right rolandic operculum, right postcentral gyrus, and right superior temporal gyrus. In the internetwork comparison, DON patients showed significantly increased FNC between the left frontoparietal network (FPN‐L) and ventral attention network (VAN). Additionally, in the intranetwork comparison, connectivity between the left medial superior frontal gyrus (MSFG) of the default network (DMN) and left putamen of auditory network was decreased in the DON group.

**Conclusion:**

DON patients altered node properties and connectivity in the DMN, auditory network, FPN‐L, and VAN. These results provide evidence of the involvement of specific brain networks in the pathophysiology of DON.

## Graphical abstract

Using independent component analysis and graph theoretical analysis to determine how brain networks altered in patients with diabetic optic neuropathy.

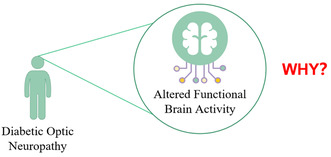

## 1 INTRODUCTION

Diabetes is a chronic disease characterized by high blood sugar levels that has increased in prevalence in the last decade, such that it now constitutes a substantial global health burden. Long‐term blood glucose instability can cause tissue and organ damage and dysfunction, and patients are at risk of a variety of complications involving the eyes, kidneys, blood vessels, and nerves. Diabetic retinopathy and diabetic macular edema are the most common ocular complications, but diabetic neuropathy can also occur,^1^ although it is frequently overlooked because of its clinical heterogeneity and difficulty of diagnosis.^2^

Diabetic neuropathy is characterized by neovascularization of the optic disk, ischemic optic neuropathy, and fundus changes that can affect the central nervous system along with peripheral and sensory nerves.^2^ Although the pathogenesis of DON is not fully understood, it is thought to be caused by a combination of optic nerve sensitivity and metabolic dysregulation in patients with diabetes. The optic nerve is particularly sensitive to hypoxia, ischemia, and metabolic disorders and diabetes can increase vascular permeability near the optic nerve in the nervous system and induce abnormalities in blood components and vascular structure in the circulatory system that result in insufficient blood and O_2_ supply to the nerve, thereby disrupting metabolism in the optic nerve.^3^, ^4^

**FIGURE 1:**
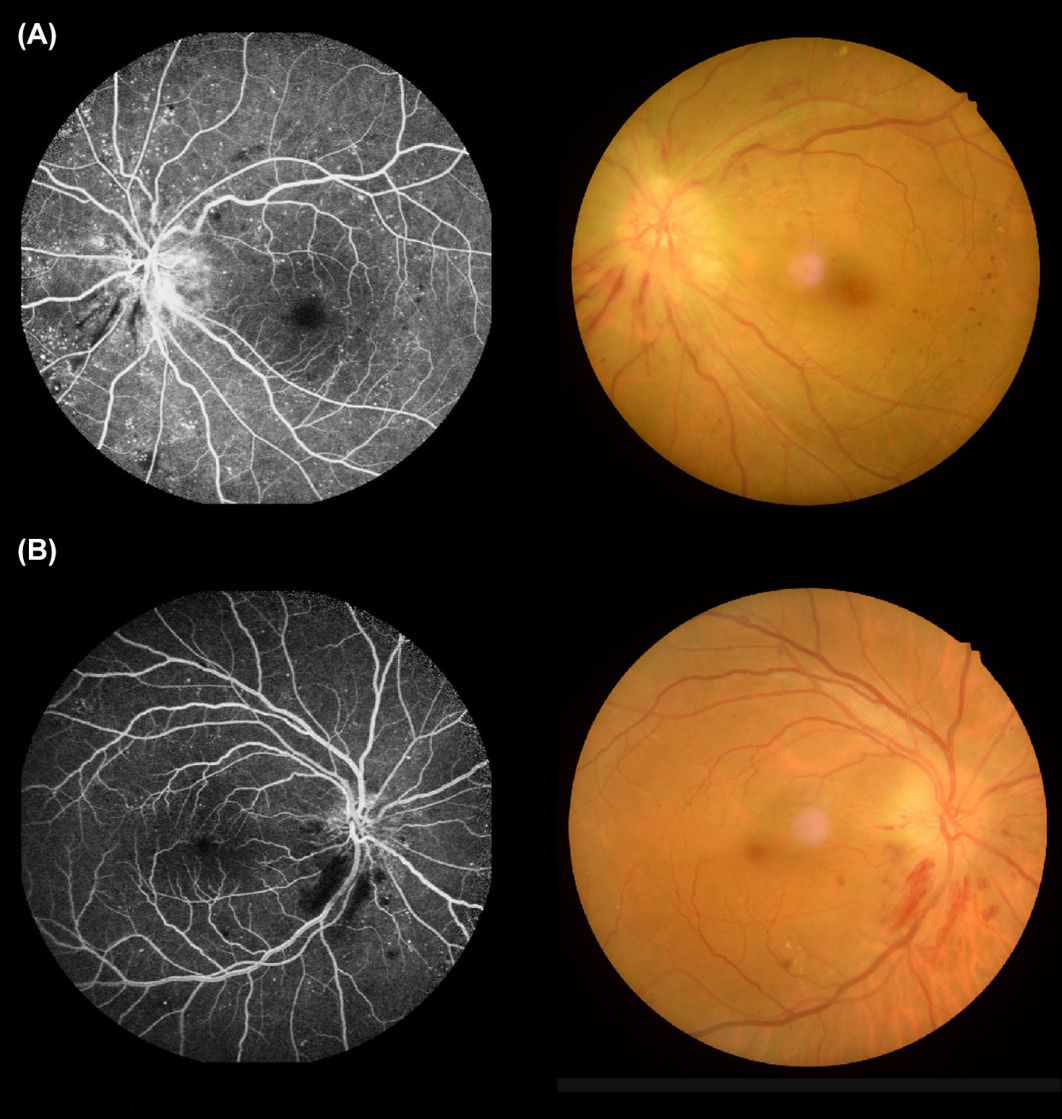
The fluorescein fundus angiography imaging of DON patients. Figure 1A, B indicate the imaging of OS and OG, respectively. OG, Right eyes; OS, Left eyes.

DON can be classified into 4 types according to clinical symptoms and etiology: optic disk neovascularization, anterior ischemic optic neuropathy, diabetic papillitis, and Wolfram syndrome.^5^ DON is usually detected by fluorescein fundus angiography (Figure 1). However, as this method requires sodium fluorescein as a contrast agent it is not suitable for diabetes patients with severe heart, liver, or kidney disease or with allergies to contrast media, making it difficult to observe the progression of DON in this population.

The human brain comprises diverse functional networks that exhibit spontaneous activity.^6^, ^7^, ^8^

Resting‐state functional magnetic resonance imaging (rs‐fMRI) is a noninvasive imaging modality that can be used to measure low‐frequency fluctuations in O_2_ level‐dependent signals in the brain.^9^ Rs‐fMRI is a standard technique in clinical diagnosis and research and is used to study psychiatric and neurologic disorders^10^, ^11^; it can also be used to examine changes in visual pathways and related brain regions in DON, providing insight into the pathogenesis and pathophysiology of this condition.

Graph theory is a method of measuring relationships between variables that can be used to describe the overall connectivity between brain networks comprising nodes (associated with a specific region) and edges (connections between pairs of regions).^12^, ^13^, ^14^ Correlations between regions reflect connectedness; in the N × N correlation matrix, each row or column corresponds to a brain region in the segment. Global and node network topology metrics such as path length (L_p_), modularity, and efficiency can be calculated from the matrix.^15^ Graph theory analysis has been applied to the study of pathogenic mechanisms underlying Alzheimer's disease (AD),^16^ schizophrenia,^17^ and epilepsy.^18^

Independent component analysis (ICA) is a statistical method for detecting hidden temporal and spatial features in brain imaging data.^19^ No prior knowledge is required for ICA, and all activated components of the results are statistically independent of each other. ICA can automatically identify functional networks of the brain from rs‐fMRI data and has been used in the study of diseases such as schizophrenia,^20^ AD,^21^ and autism.^22^

In the present study, we assessed global functional and regional nodal properties and functional connectivity (FC) of brain networks in patients with DON by applying graph theoretical analysis and ICA, respectively, to rs‐fMRI data.

## 2 MATERIALS AND METHODS

### 2.1 Subjects

This current study enrolled 23 DON patients and 41 HCs from the Department of Ophthalmology at the First Affiliated Hospital of Nanchang University in Jiangxi Province, China.

Inclusion standards for DON were as follows: (1) medical history of diabetes mellitus; (2) indication of ischemic optic neuropathy, optic atrophy, optic disk edema, or neovascularization; (3) FFA examination revealed abnormal fluorescence in the optic nipple; (4) with usual clinical manifestations like the basis of visual impairment was clinically observed and the degree of vision deterioration varies, patients have increased physiological blind spots or simply have lower visual acuity; and (5) no systemic diseases. Additionally, 41 healthy controls (HCs) were gathered, who met the following requirements: (1) no past medical history of eye problems; (2) no previous history of mental or neurological disorders; and (3) standard MRI brain parenchyma, with no contraindications for MRI scans, for instance, pacemakers or other metal implants.

### 2.2 MRI parameter

All magnetic resonance imaging (MRI) tests were performed on the Siemens Trio, a 3 Tesla MRI scanner with an eight‐channel head coil. All individuals stayed awake and breathe smoothly with closed eyes throughout the scan. A total of 240 functional images, firstly, scan the even layers and then the odd layers. The specific scanning parameters that were employed are stated below: (1) scanning number of plies = 30; (2) acquisition matrix = 64 × 64; (3) field of view = 220 × 220 mm; (4) thickness = 4.0 mm; (5) gap = 1.2 mm; (6) repetition time = 2000 msec; (7) flip angle = 90°; and (8) echo time = 30 msec and 29 axial.

### 2.3 The preprocessing of fMRI data

Functional MRI Data were preprocessed by SPM12 (http://www.fil.ion.ucl.ac.uk/spm) and DPABI (http://rfmri.org/dpabi). The detailed preprocessing information before GTA was as follows: (1) delete the first ten time points; (2) time level correction; (3) head motion correction, head motion parameters more than 2 mm during scanning were excluded; (4) EPI registration; (5) linear trends were removed within BOLD; (6) the time series of every voxel generated the covariates containing the white matter signal, cerebrospinal fluid signal, and head motion friston 24; and (7) using a temporal band‐pass to filter all smoothed images (0.01 Hz‐0.08 Hz). The detailed preprocessing information before ICA have 5 steps, which the first 4 steps are same as above. The 5th step of fMRI data prepossessing before ICA is that with a Gaussian kernel of 6 × 6 × 6 mm^3^ full width at half maximum, and all datasets were smoothed. After pretreatment, we left 41 healthy controls and 23 patients for follow‐up analysis.

### 2.4 Brain network establishment in graph theoretical analysis

We used GRETNA, a toolbox for imaging the connectome, to build functional brain networks.^23^ The whole brain is divided into 90 regions of interest after using the AAL90 template to define the network nodes, each of them signifying a network node. The time series for each of the 90 regions was extracted from the preprocessed data. The mean time series of all 90 regions were calculated to get Pearson's correlation coefficients, which is for each pair of regions, and the data were transformed into z‐values using Fisher's Z transformation. Sparsity was used as a range of correlation coefficient thresholds for the network construction.^24^ Graph theoretical analysis was used to evaluate the topological and organizational characteristics of the entire brain. The functional network's sparsity was set to range from 0.1 to 0.34 (in 0.01 steps), to ensure that the network has both small‐world attributes and sparse characteristics. We calculate the network attributes of each sparsity, including small‐worldness (σ), normalized characteristic path length (λ), normalized clustering coefficient (γ), clustering coefficient (C_p_), characteristic path length (L_p_), global efficiency (E_glob_), local efficiency (E_loc_), degree centrality (DC), nodal efficiency (NE), and nodal local efficiency (NLE). The area of the curve under each index was calculated, and finally, the area under the curve was statistically compared between groups.

### 2.5 Independent component analysis in functional network connectivity

**FIGURE 2:**
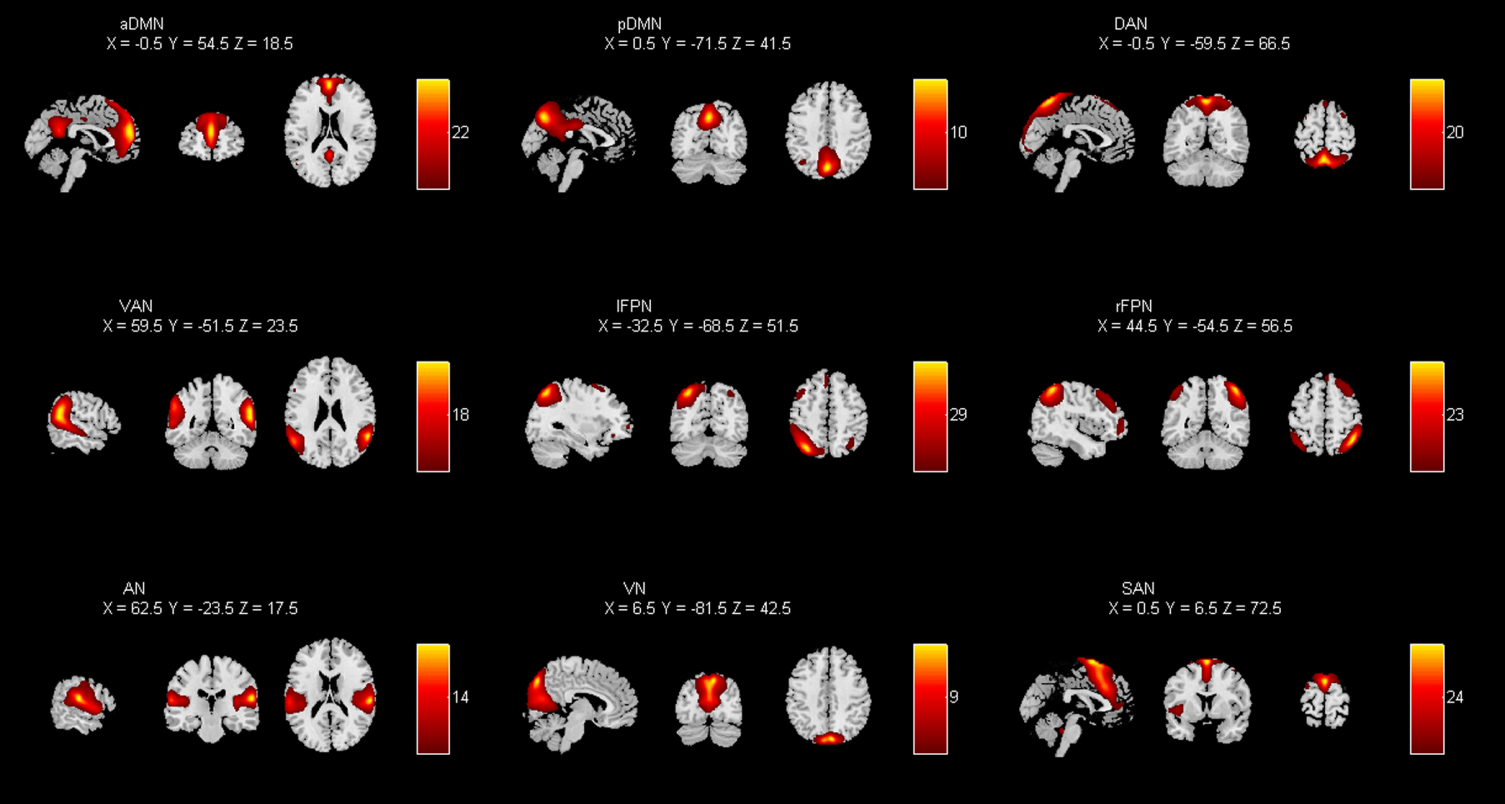
Spatial maps of 9 selected independent components. aDMN, anterior default mode network; AN, auditory network; DAN, dorsal attention network; lFPN, left frontoparietal network; pDMN, posterior default mode network; rFPN, right frontoparietal network; SAN, salience network; VAN, ventral attention network; VN, visual network.

With the help of the GIFT toolbox (http://mialab.mrn.org/software/gift/), ICA was used to segment the preprocessed fMRI data. According to the minimum description length (MDL) criterion, the software automatically estimates the independent component number to be 33. Before ICA decomposition, principal component analysis (PCA) was used to minimize the dimensionality of the data. Firstly, PCA was applied to lessen the subject‐specific data into 50 major components. After dimensionality reduction, individual data are spliced according to time points to group‐level data. Principal component analysis was performed on group‐level data, and 33 principal components were retained. The infomax algorithm was repeated 20 times in ICASSO (http://research.ics.tkk.fi/ica/icasso/) to ensure the estimation stability. Finally, using the GICA back reconstruction method, participant‐specific spatial maps and time sequences were generated. Selecting ingredients from the results based on experience: (1) Functional networks that had active spikes should be in gray matter and showed low spatial overlap with cerebrospinal fluid, white matter, and ventricles, and (2) time sequences of functional networks exhibited primarily low‐frequency power. This selection process resulted in 9 functional networks out of 33 independent components (Figure 2): anterior and posterior default mode networks (aDMN and pDMN); dorsal and ventral attention networks (DAN and VAN); left and right frontoparietal networks (FPN‐L and FPN‐R); auditory network (AN); visual networks (VN); and salience networks (SAN).

### 2.6 Statistical analysis

SPSS 20.0 software (SPSS) was utilized for the demographic data differences between DON patients and healthy controls. An independent sample *t*‐test and chi‐square test were used in comparisons between DON patients and HCs. It is set that the significance level at *p* < 0.05 and values are expressed as the mean ± standard deviations. Before the two‐sample *t*‐test, all data detected by these four methods – the Anderson–Darling test, the D'Agostino–Pearson test, the Shapiro–Wilk test, and the Kolmogorov–Smirnov test to check normality and lognormality – if some data do not meet acquirements, then the data detected by Mann–Whitney test, rather than unpaired *t*‐test. The two‐sample *t*‐test was used to test for differences between the two groups at *p* < 0.05 (FDR corrected), with age and gender as covariates.

## 3 RESULTS

### 3.1 Demographics

The demographic and clinical characteristics of the study population are shown in Table 1. There were no differences in age, sex, or body weight between patients with DON and healthy control (HC) subjects (*p* > 0.05).

**TABLE 1:** Demographic and clinical characteristics between DON and HCs.

Characteristics	HCs	DON	Statistics	*p* values
Male/female	14\27	7\16	*χ* ^2^ = 0.092	0.762
Age (years)	55.87 ± 6.74	56.89 ± 5.63	−0.397	0.693
Weight (kg)	66.89 ± 8.86	65.48 ± 7.21	0.314	0.897
Handedness	41R	23R	N/A	N/A
Duration of DON (days)	N/A	45.52 ± 5.64	N/A	N/A
Best‐corrected VA, right	1.05 ± 0.25	0.32 ± 0.12	−4.053	0.021
Best‐corrected VA, left	1.05 ± 0.20	0.25 ± 0.16	−3.366	0.018

Note: The data are shown as the mean values ± standard deviations. The *p* value was obtained by the chi‐squared test and the independent *t*‐test.

Abbreviations: BMI, body mass index; DON, diabetic optic neuropathy; HCs, healthy controls; NA, not applicable; rs‐fMRI, resting‐state functional magnetic resonance; VA, visual acuity.

### 3.2 Graph theoretical analysis of global functional properties

**FIGURE 3:**
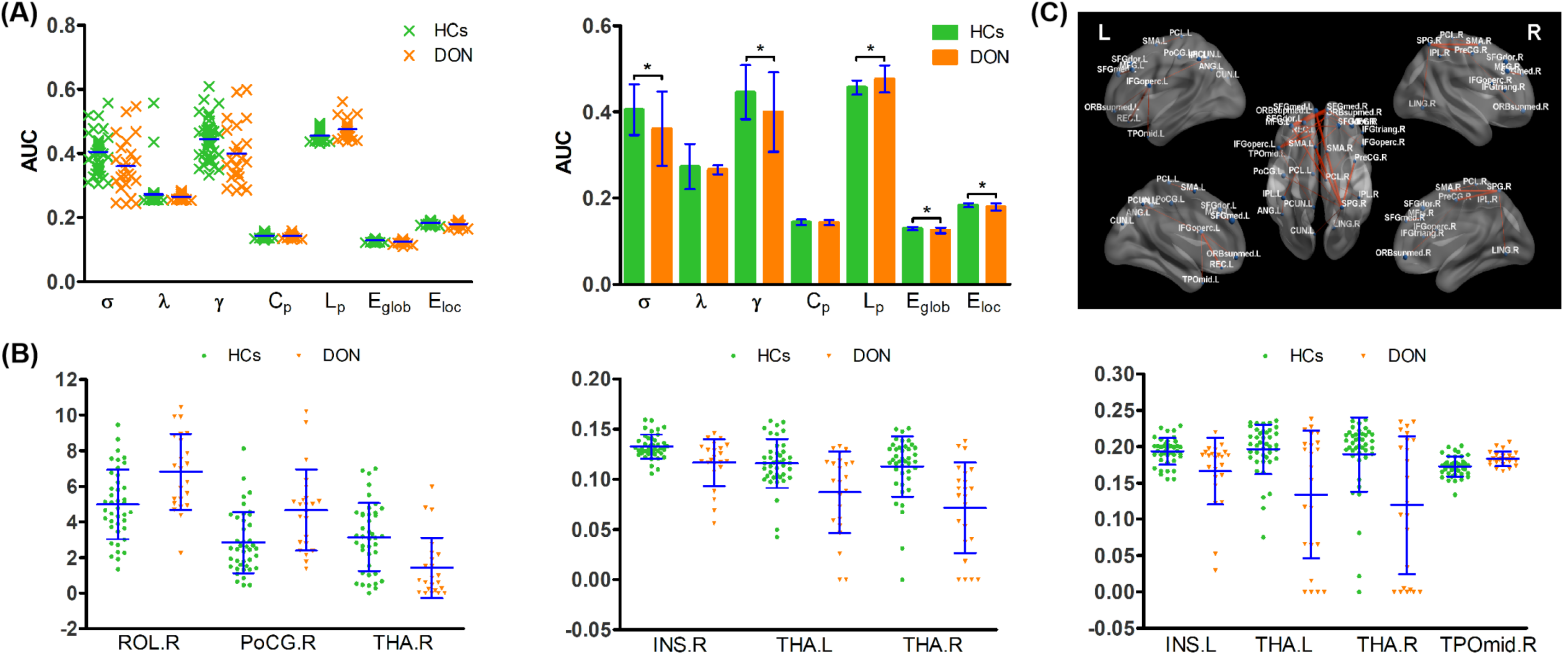
(A) The global functional properties. DON patients had significantly lower values of σ (*p* = 0.0185) and γ (*p* = 0.0211), global efficiency (E_glob_) (*p* = 0.0021), and local efficiency (E_loc_) (*p* = 0.0164), but higher values of L_p_ (*p* = 0.0016). There were no significant differences between groups in λ or clustering coefficient (C_p_) (*p* > 0.05). The values represent the AUC values (mean values ± standard deviations). Significant at *p < 0.05, independent *t*‐test. C_p_, clustering coefficient; DON, diabetic optic neuropathy; E_glob_, global efficiency; E_loc_, local efficiency; HCs, healthy controls; L_p_, characteristic path length. (B) The nodal properties between DON and HCs. The values represent the AUC values (mean values ± standard deviations). Significant differences between DON and HCs were corrected for false discovery rate at *p* < 0.05. DON, diabetic optic neuropathy; HCs, healthy controls; L, left; R, right. (C) The functional connections of connectivity networks. The differences between the DON and HCs were evaluated by two‐sample test in connected edges (edge *p* < 0.001, component *p* < 0.05) with 10,000 permutations.

The choice of threshold affects the topologic characteristics of neural networks. In the sparse range of 0.1–0.34 (in increments of 0.01), the DON and HC groups had higher clustering coefficients and shorter average L_p_ compared with matched random networks, indicating that the resting brain functional networks of both groups had small‐world topology (Table 2). However, the 2 groups showed differences in small‐world parameters and nodal properties (Figure 3A and Table 3): Patients with DON had significantly lower values of small‐worldness (σ) (*p* = 0.0185), normalized clustering coefficient (γ) (*p* = 0.0211), and global efficiency (E_glob_) (*p* = 0.0021), and local efficiency (E_loc_) (*p* = 0.0164), but higher values of L_p_ (*p* = 0.0016).There were no significant differences between groups in λ or clustering coefficient (C_p_) (*p* > 0.05).

**TABLE 2:** The global properties between DON and HCs.

	DON	HCs	*t*	Mann Whitney *U*	*p*‐Values
Sigma	0.361 ± 0.086	0.405 ± 0.059	−2.419		0.0185*
Lambda	0.266 ± 0.010	0.262 ± 0.006	\	424.0	0.5108
Gamma	0.400 ± 0.092	0.445 ± 0.062	−2.367		0.0211*
C_p_	0.143 ± 0.006	0.144 ± 0.006	−0.601		0.5503
L_p_	0.476 ± 0.031	0.456 ± 0.016	3.296		0.0016*
E_glob_	0.125 ± 0.006	0.129 ± 0.004	−3.209		0.0021*
E_loc_	0.180 ± 0.008	0.184 ± 0.005	−2.467		0.0164*

*Note*: The values represent the AUC values (mean values ± standard deviations). Significant at *p < 0.05, independent *t*‐test.

Abbreviations: C_p_, clustering coefficient; DON, diabetic optic neuropathy; E_glob_, global efficiency; E_loc_, local efficiency; HCs, healthy controls; L_p_, characteristic path length.

**TABLE 3:** The nodal properties between DON and HCs.

Brain regions	*p* value		
	Nodal degree	Nodal efficiency	Nodal local efficiency
DON > HC			
Rolandic operculum_R	0.001	–	–
Postcentral gyrus_R	0.001	–	–
Superior temporal gyrus _R	–	–	0.002
DON < HC			
Insula_L	–	–	0.001
Insula_R	–	0.000	–
Thalamus_L	–	0.001	0.000
Thalamus_R	0.001	0.000	0.000

Note: The values represent the AUC values (mean values ± standard deviations). Significant differences between DON and HCs were corrected for false discovery rate at *p* < 0.05.

Abbreviations: DON, diabetic optic neuropathy; HCs, healthy controls; L, left; R, right.

In terms of nodal properties, patients with DON showed increased nodal degree in the right rolandic operculum (ROL‐R), right postcentral gyrus (POCG‐R), and right superior temporal gyrus (STG‐R) and decreased values in the right thalamus compared with HCs. In terms of nodal efficiency (NE), the DON group showed reduced values in the right insula and thalamus; additionally, nodal local efficiency (NLE) was increased in ROL‐R and POCG‐R and decreased in the right insula (Figure 3B and Table 3).

### 3.3 Connected network analysis

We used network‐based statistics to compare connectivity networks between patients with DON and HCs. We used age as a covariate and used the two‐sample *t*‐test to evaluate differences in connected edges between the 2 groups (edge *p* < 0.001, component *p* < 0.05) with 10,000 permutations. The functional connections of connectivity networks in patients with DON and HCs are shown in Figure 3C.

### 3.4 Pairwise correlation patterns

**FIGURE 4:**
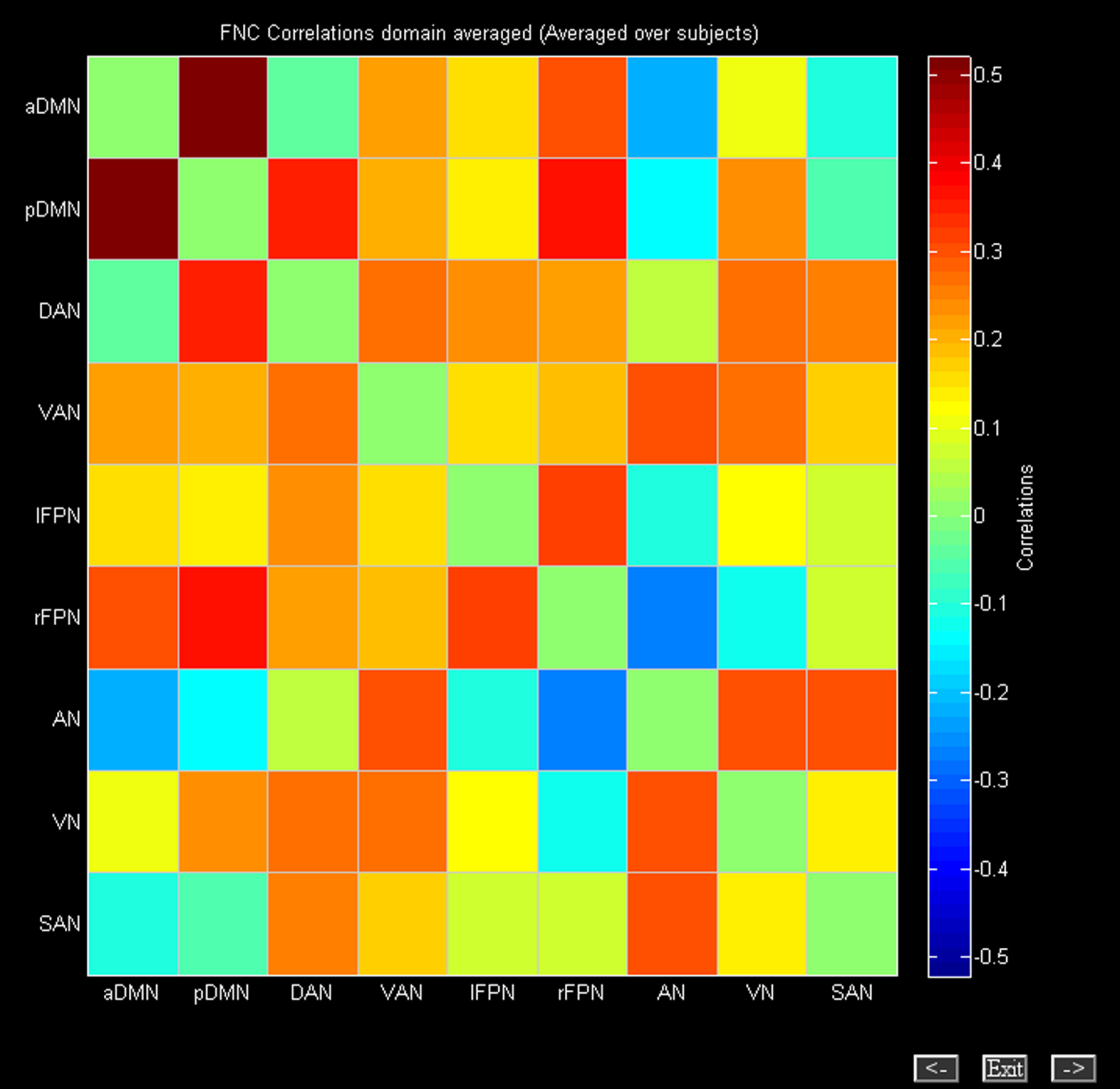
Internetwork functional connectivity matrix. The pairwise correlation between functional networks was averaged across subjects. Hot colors indicate positive functional connectivity, and cool colors indicate negative functional connectivity. aDMN, anterior default mode network; AN, auditory network; DAN, dorsal attention network; lFPN, left frontoparietal network; pDMN, posterior default mode network; rFPN, right frontoparietal network; SAN, salience network; VAN, ventral attention network; VN, visual network.

We calculated Pearson's correlation coefficients of 9 functional networks (anterior default mode network [aDMN], posterior DMN [pDMN], dorsal attention network [DAN], ventral AN [VAN], left frontal–parietal network (FPN‐L), right FPN (FPN‐R), auditory network, visual network, and salience network) between DON patients and HCs to construct the functional network connectivity (FNC) matrix (Figure 4).

### 3.5 Inter‐ and intranetwork FC analysis

**FIGURE 5:**
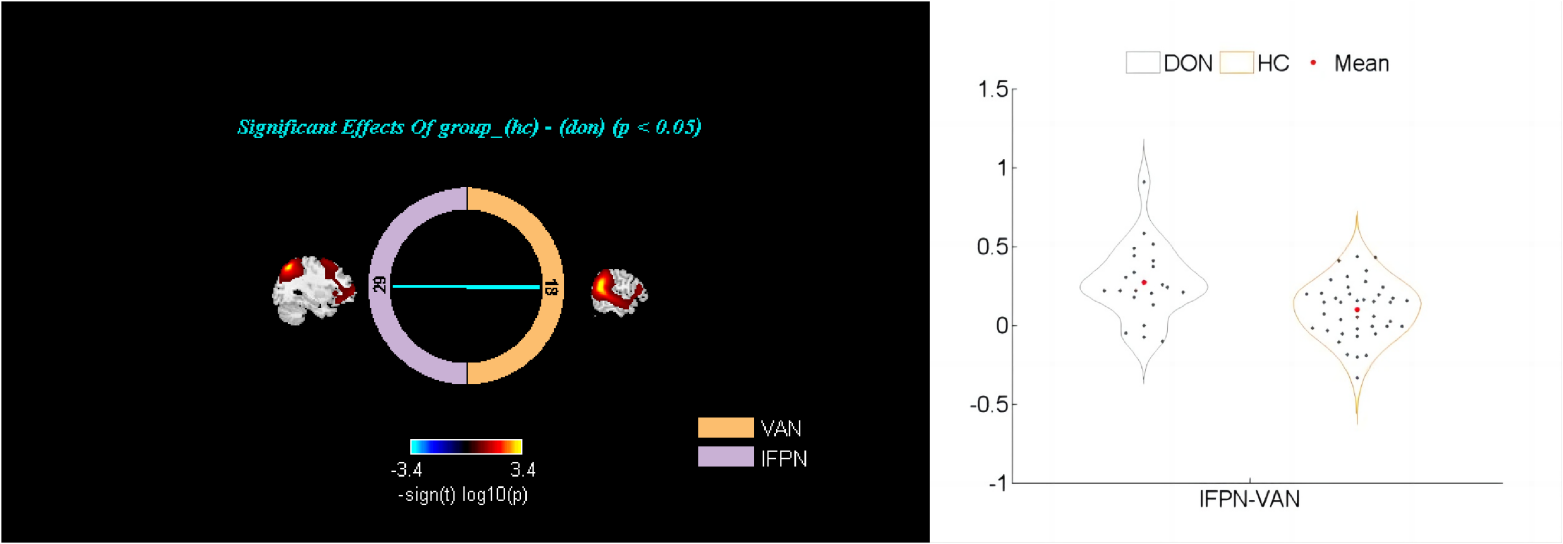
The internetwork FC analysis. The results showed that FC between the FPN‐L and VAN was significantly higher in the DON group than in the HC group, which were corrected for multiple comparisons with a false discovery rate‐adjusted significance level of *p* < 0.05.

**FIGURE 6:**
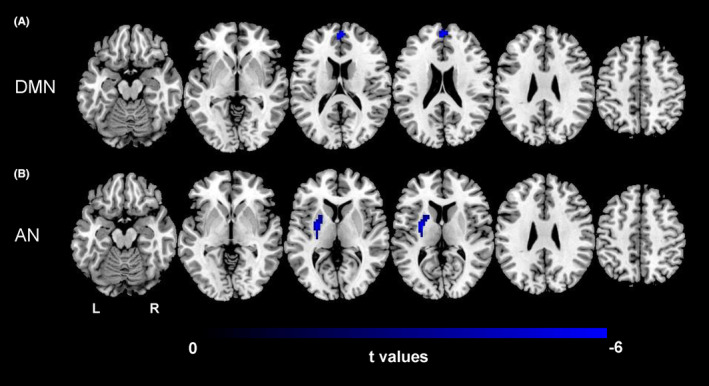
The intranetwork FC analysis. The left medial superior frontal gyrus (MSFG) of the DMN default network (extending to the right MSFG) (Figure 6A) and left putamen of the auditory network (Figure 6B) were lower in patients with DON than in HCs.

With sex and age as covariates, we used the two‐sample *t*‐test to compare inter‐ and intranetwork FC between the DON and HC groups. The results were corrected for multiple comparisons with a false discovery rate‐adjusted significance level of *p* < 0.05 (Figure 5). The results showed that FC between the FPN‐L and VAN was significantly higher in the DON group than in the HC group. Additionally, the left medial superior frontal gyrus (MSFG) of the DMN default network (extending to the right MSFG) (Figure 6A) and left putamen of the auditory network (Figure 6B) were lower in patients with DON than in HCs.

## 4 DISCUSSION

### 4.1 Comparative graph theoretical analysis

The brain is a complex system with distinct functional areas for processing (i.e., separating and integrating) different types of information.^25^ The information flow between interconnected brain regions is the basis of all cognitive processes. Previous studies have shown that patients with diabetic retinopathy exhibit abnormal neuronal synchronization in many areas of the brain, which is consistent with cognitive and visual functional deficits.^26^ Small‐world topology is characterized by the dense local clustering or cliquishness of connections, with only a small number of long‐range connections; this is used to model the architecture of anatomical and functional brain networks.^27^

The results of the present study show that whole‐brain networks in both patients with DON and HC subjects showed efficient small‐world topology, consistent with previous reports.^28^, ^29^ Nonetheless, there were differences between the 2 groups in small‐world parameters and nodal properties. Changes in global network properties in DON patients suggested a breakdown of the normal balance in functional networks caused by a decrease in long‐distance functional connections mainly involving the auditory network and DMN.

Compared with HCs, the brain networks of DON patients had increased L_p_ value but decreased in E_glob_ and E_loc_.

Cp and E_loc_ measure the transmission capacity of local information in the brain network and are closely related to the separation and transmission of information.^30^ E_glob_ reflects the convenience of information exchange between network nodes. Lp reflects a path with high information transmission efficiency, and both measure the information transmission capability of the brain network at the global level. Previous studies have shown that the decrease of the Eg of the brain functional network is related to the decline of cognitive function and memory, and some studies have also confirmed that cognitive impairment is related to diabetes mellitus.^31^, ^32^ Therefore, the decrease in the Eg of DON patients in this study may be related to the cognitive decline of these patients. These changes may be caused by the changes in the internal structure and network connectivity of the brain functional network of DON patients.

### 4.2 Comparison of nodal properties

Compared with HCs, degree centrality (DC), NE, and NLE were significantly decreased in the right thalamus of the DON group. DC reflects the significance of nodes in the brain network, whereas NE and NLE are related to local information integration and transmission capacity.^29^, ^30^

The thalamus has structural and functional connections with multiple cortical regions and participates in the filtering of sensory information from receptors, which is then projected to target cortical regions. In this manner, the thalamus plays an important role in language, emotion processing, and executive functions.^33^ DC of bilateral thalamus was shown to be decreased in chronic heroin users, which was associated with impairments in planning, memory, observation, and attention.^32^ It has also been reported that damage to structural connections in the thalamus decreased information transmission efficiency and integration and was related to the clinical symptoms of autism spectrum disorder.^34^ Another study illustrated those nonpsychotic relatives, in comparison with healthy controls, also displayed greater nodal degree centrality properties in the left posterior cingulate cortex and THA‐L, which indicated an improved connection between the node and other nodes in the neural network architecture.^35^ In agreement with these previous studies, our findings suggest that the thalamus white matter network is impaired in patients with DON, which may lead to deficits in language and emotion processing and executive functions.

The inferior frontal gyrus (IFG) of the insula is involved in semantic and speech processing and language generation. Functional imaging studies have shown that the insular cortex plays a role in essential auditory functions including tuning into novel auditory stimuli and allocating auditory attention^36^; bilateral insula damage may lead to auditory agnosia. There is also evidence from clinical studies for the involvement of the insula in protopathic sensibility.^37^ In this study, we found that the NE and NLE of the insula IFG node were decreased in the DON group, as previously reported.^38^, ^39^ It is possible that DON is associated with decreased insula activity, which may have negative effects on language and auditory functions in patients.

The STG is related to auditory and language processing; damage to this structure can cause auditory hallucinations and thought disorder. Reduced STG and left amygdala volume was shown to be correlated with greater severity of hallucinations.^40^ Another study showed that patients with external space hallucinations had a significantly smaller volume of white matter in the voxel cluster located in the right STG compared with patients with internal space hallucinations,^41^ suggesting that differences in hallucination symptoms are related to specific areas of the STG. In the current study, the increased NLE in the STG‐R of DON patients may be a compensatory response to disordered visual and verbal information processing.

The POCG is in the somatosensory cortex and participates in sensory information processing and emotion regulation.^42^ Voxel‐based DC is an index of node information transmission capacity and reflects the importance of a region in the brain network hub. We found that the DC in the POCG‐R was increased in patients with DON, indicating that there were more connections between the POCG‐R and other brain regions compared with HCs along with a corresponding increase in information transmission capacity. We speculate that the increased DC in the POCG‐R of DON patients reflect network reorganization to compensate for a dysfunction of sensory information processing and emotion regulation.

The ROL is located on the precentral and postcentral gyri and contributes to emotion processing and gustatory and visceral sensation.^43^ The increase in DC in the ROL‐R of patients with DON may also be a compensatory mechanism and indicates that the ROL is perturbed in DON.

### 4.3 ICA of FNC

ICA is frequently applied to brain imaging data as it does not require pre‐assumptions and can be used to assess correlations between blood oxygen level‐dependent signals in whole‐brain voxel attributes. Resting‐state functional networks are thought to facilitate efficient inter‐ and intranetwork information processing and ensure an optimal balance between functional integration and separation.^39^, ^40^, ^41^, ^44^

### 4.4 Intra‐ and internetwork comparisons of FNC

The FPN‐L is a language‐ and cognition‐related brain network. FNC between the FPN‐L and the right inferior, medial, and middle parts of the frontal cortex was shown to be reduced in patients with aphasia, implying that impaired language comprehension is related to a decrease in functional connections between language centers and other regions.^43^

VAN is a component of the orbitofrontal cortex and middle temporal gyrus that is highly sensitive to insulin.^45^ Individuals with prediabetes show stronger connectivity between the VAN and visual or sensorimotor network.^46^ Enhancement of FNC involving the DMN and frontoparietal network has also been observed in patients with traumatic brain injury during the recovery period.^47^ We propose that this is a compensatory mechanism that allows adaptation to pathologic changes.^48^ In this study, the FNC between FPN‐L and VAN was enhanced in DON patients compared with HCs. Thus, increased FNC may indicate deficits in connectivity and compensatory activation between attention and language/cognition centers and other brain areas.

Certain cortical regions of the human brain are co‐activated in the resting state and form a connected functional network known as the DMN that is involved in emotional cognition. Altered connectivity in the DMN have been linked to mental disorders.^49^ For example, patients with depression show increased connectivity in the DMN.^50^, ^51^ FNC was significantly decreased in the prefrontal region of the DMN and increased in the hippocampus in glioma patients, which was associated with tumor grade.^52^ The results of our study indicate that the left MSFG (extending to the right MSFG) of the DMN was lower in the DON group than in HCs, suggesting that decreased FNC in the DMN is related to dysfunctional emotional cognition in DON. The lateral STG, planum polar, planum temporal, Heschl's gyri, and posterior insula are components of the auditory network^53^ that also participate in the integration and convergent processing of information from other sensory modalities.^54^ The auditory network also enables interaction with the environment through processing of auditory information including pitch,^55^ tone,^56^ and speech.^57^ Schizophrenia patients with auditory hallucinations show greater co‐activation in the right auditory cortex and bilateral insula of the auditory network than patients without auditory hallucinations^58^; and compared with healthy individuals, schizophrenia patients have lower FC in the left insula and cingulate gyrus of the auditory network.^58^ We found that FNC was significantly decreased in the left putamen of the auditory network in the DON group, which may be associated with functional impairment resulting from a reduction in auditory network interactions with the environment.

## 5 CONCLUSION

In this study, by analyzing the data from rs‐fMRI, we found DON patients in small‐world network properties, node properties exhibit differences by graph theoretical analysis and DON patients showed altered connectivity in the DMN, AN, FPN‐L, and VAN.

This study may be applicable to DON patients with contraindications to fluorescein fundus angiography, but we need more experimental data to support the development of precise criteria to assist diagnosis. For instance, does this indirect analysis of rs‐fMRI have universality – whether influenced by region or ethnicity? In the future, we plan to conduct more comprehensive experiments to gradually explore the pathophysiological mechanism of DON and formulate a good diagnosis and treatment policy.

## CONFLICT OF INTEREST STATEMENT

This study did not receive any industrial support. The authors have no competing interests to declare regarding this study.

## CONSENT TO PARTICIPATE

All subjects were notified of the objectives and content of the study and latent risks, and then signed informed consent forms to participate.

## Data Availability

The datasets used and/or analyzed during the present study are available from the corresponding author upon reasonable request.
